# Impact of the COVID-19 pandemic on hospital admission rates for arterial hypertension and coronary heart disease: a German database study

**DOI:** 10.3389/fcvm.2024.1333749

**Published:** 2024-05-14

**Authors:** Benjamin Sasko, Marios Matiakis, Felix S. Seibert, Nikolaos Pagonas, Hans-Jörg Hippe, Nina Babel, Christian Ukena, Timm H. Westhoff

**Affiliations:** ^1^ Medical Department II, Marien Hospital Herne, Ruhr-University of Bochum, Herne, Germany; ^2^ Medical Department I, Marien Hospital Herne, Ruhr-University of Bochum, Herne, Germany; ^3^ Department of Cardiology, University Hospital Ruppin-Brandenburg, Medical School Theodor Fontane, Neuruppin, Germany; ^4^ Faculty of Health Sciences, Joint Faculty of the Brandenburg University of Technology Cottbus—Senftenberg, The (MHB) Theodor Fontane and the University of Potsdam, Potsdam, Germany; ^5^ Department of Cardiology, Marien Hospital Witten, Witten, Germany; ^6^ Center for Translational Medicine, University Hospital Marien Hospital Herne, Ruhr University Bochum, Herne, Germany

**Keywords:** endothelial dysfunction, COVID-19, coronary artery disease, acute myocardial infarction, hospitalisation

## Abstract

**Background:**

During the SARS-CoV-2 pandemic it was speculated that the virus might be associated with a persistent increase of cardiovascular risk. The present study compares pre- and post-pandemic hospital admission rates for hypertension and coronary artery disease.

**Methods:**

Systematic multicentric retrospective cohort analysis of 57.795 hospital admissions in an urban region in Germany during two different periods (pre-pandemic 01–06/2019 vs. post-pandemic era 01–06/2023). Information on hospital admissions for arterial hypertension, chronic coronary syndrome, unstable angina pectoris and acute myocardial infarction were extracted from the hospitals data systems. Additionally, six comorbidities and performed coronary interventions were monitored.

**Results:**

Compared to the pre-pandemic era, there was no increase in hospitalizations for arterial hypertension (516 vs. 483, −6.8%, *p* = 0.07) or myocardial infarction (487 vs. 349, −23.8%, *p* < 0.001), but the total number of patient admissions with chest pain as the presenting symptom increased (chronic coronary syndrome: 759 vs. 943, +24.2%, *p* < 0.001; unstable angina pectoris: 270 vs. 451, +67.0%, *p* < 0.001). At the same time, the number of performed coronary angiographies increased, but less patients underwent percutaneous interventions. Patients admitted with chest pain in the post-pandemic era were in general healthier with less comorbidities.

**Conclusion:**

The present multicenter cohort study found no evidence for an increase in hospitalizations for arterial hypertension or coronary artery disease after the end of the pandemic. However, further studies with larger sample sizes are needed to confirm our results.

## Introduction

During the global coronavirus disease 2019 (COVID-19) outbreak 770 million worldwide confirmed infections caused by severe acute respiratory syndrome coronavirus 2 (SARS-CoV-2) have been reported by the World Health Organization (1). Beyond respiratory symptoms, COVID-19 is associated with systemic endothelial damage leading to micro- and macrovascular thrombotic events. Macrovascular events comprise both venous thrombembolism and arterial thrombotic events including myocardial infarction, stroke, and limb ischemia (2). SARS-CoV-2 can cause ubiquitous endothelial damage both by infection of endothelial cells or indirectly via cytokine storm (3) resulting in endothelial dysfunction (endotheliitis, endothelialitis and endotheliopathy) and multi-organ injury.

Endothelial damage is a crucial prerequisite for the development of atherosclerosis and contributes to arterial hypertension. Therefore, the above mentioned findings raised serious concerns on a potentially persistently increased cardiovascular risk leading to augmented morbidity and mortality. To date, it remains elusive, whether there is an endothelial reconstitution ad integrum after an acute episode of COVID-19 or whether there is a residual long-lasting endothelial damage. If so, this could indeed lead to increased prevalence of arterial hypertension and increased coronary events. It is not able to perform a cohort study on endothelial function before and after the pandemic. It is possible, however, to investigate existing surrogate parameters of cardiovascular risk in regional cohorts. In the present multicenter cohort study, we therefore compare hospital admission rates for hypertensive emergency, angina pectoris, acute coronary syndrome and coronary revascularization before and after the pandemic as surrogate markers of cardiovascular risk in three large nearby hospitals in an urban region in Germany.

## Methods

Three nearby tertiary hospitals in Germany participated in this analysis and supplied anonymous patient data. We performed a systematic multicentric retrospective analysis using an electronic data extraction approach to identify subjects who were admitted for arterial hypertension, chronic coronary syndrome (CCS), unstable angina pectoris (UAP) and acute myocardial infarction (AMI). The study period, extended from 1 January 2023 to 30 June 2023 (post-COVID-19 pandemic), while the control period was defined as the corresponding time period four years earlier, from 1 January 2019 to 30 June 2019 (pre-COVID-19 pandemic). Due to the retrospective nature of the study with solely anonymous data, ethic approval was waived by the local ethics committee.

## Data extraction

Data of hospital admissions were extracted from the hospitals’ digital information systems. Supplementary Table S1 in the supplements provides an overview on the disease codes for hypertensive emergency, unstable angina pectoris, chronic coronary syndrome and acute myocardial infarction (STEMI and Non-STEMI). Admissions were identified by using the International Diseases Coding 10. Details can be found in Supplementary Table S1.

Additionally, six comorbidities were extracted as secondary diagnosis: arterial hypertension, diabetes mellitus, chronic kidney disease, hypercholesterinemia, congestive heart failure and atrial fibrillation. Details can be found in Supplementary Table S2.

To assess the number of coronary angiography and percutaneous coronary angioplasty (PCI) performed in the observational periods, the procedural codes of performed procedures during the two periods were extracted from the hospital data system by using the German operation and procedure classification system “Operationen- und Prozedurschlüssel”, “OPS”), which serves as a coding system for operations and treatments published by the German ministry of health. Codes of coronary angiography and percutaneous intervention can be found in Supplementary Table S3.

## Statistical analysis

Descriptive analysis was performed using absolute numbers and rates of hospital admissions for arterial hypertension, CCS, UAP and AMI. Performed procedures were described as absolute numbers. The differences in numbers of admission or treatment rates before and after the pandemic were tested using *χ*^2^ test. Continuous variables were compared using Mann–Whitney *U-*test.

To analyze the hospitalization rates between the control period and the study period, we compared the admission rates of grouped weeks by using Mann–Whitney *U*-Test. Multivariate analysis using linear regression was used to assess whether potentially confounding variables exists. For that reason, we performed a multivariate analysis using a model adjusted for age and sex. In addition, diabetes mellitus and the presence of a chronic kidney disease were added as potentially confounding variables in the full model.

A two-sided *p*-value less than 0.05 was considered statistically significant in all performed analyses. All statistical analyses were performed using SPSS software (Version 23).

## Results

Hospital admissions for any cause increased by 1.315 patients from 28.240 (January to June 2019) to 29.555 (January to June 2023) (+4.7%). Emergency admissions, however, remained constant (10.034 vs. 10.109, +0.7%). Hospital admissions due to arterial hypertension tended to decrease without reaching statistical significance (516 vs. 483, −6.8%, *p* = 0.07), with a higher female proportion. Data on admissions for coronary heart disease diverged: There was a significant increase in hospital admissions due to CCS by 184 patients (759 vs. 943, +24.2%, *p* < 0.001) and UAP (270 vs. 451, +67.0%, *p* < 0.001), but a decrease in admission for AMI by 138 patients (487 vs. 349, −23.8%, *p* < 0.001). With the exception of UAP, gender distribution did not differ between the control and study period. The duration of hospitalization significantly decreased over time for all investigated diagnoses. Descriptive data are summarized in Table 1.

**Table 1:** Number of patients hospitalized during control and study period with corresponding age, gender and length of hospital stay.

Admission type				
	Control period	Study period	Difference	
Total	28.240	29.555	+1.315 (4.7%)	
Emergencies	10.034	10.109	+75 (0.7%)	
Number of admissions according to admission diagnose				
	Control period	Study period	Total difference	*p*-value
Hypertension	516 (1.8%)	483 (1.6%)	−33 (6.8%)	0.07
Female	349 (67.6%)	313 (64.8%)		0.34
Age (years)	65.4 (±15.8)	64.7 (±15.7)		0.5
Length of stay (days)	2.9 (±2.0)	2.6 (±1.8)		**<0** **.** **001**
CCS	759 (2.6%)	943 (3.2%)	+184 (24.2%)	**<0** **.** **001**
Female	221 (29.1%)	306 (32.4%)		0.13
Age (years)	69.3 (±10.3)	69.1 (±10.6)		0.8
Length of stay (days)	3.0 (±2.8)	2.5 (±2.0)		**<0** **.** **001**
UAP	270 (0.9%)	451 (1.5%)	+181 (67.0%)	**<0** **.** **001**
Female	83 (30.7%)	191 (42.4%)		**0** **.** **002**
Age (years)	67.4 (±12.3)	68.5 (±11.5)		0.28
Length of stay (days)	3.1 (±2.1)	2.7 (±1.8)		**<0** **.** **001**
NSTEMI/STEMI	487 (1.7%)	349 (1.1%)	−138 (28.3%)	**<0** **.** **001**
Female	182 (37.4%)	121 (34.7%)		0.42
Age (years)	71.2 (±13.6)	70.5 (±13.4)		0.51
Length of stay (days)	6.2 (±8.0)	4.9 (±4.6)		**0** **.** **007**

CCS, chronic coronary syndrome; UAP, unstable angina pectoris; NSTEMI, non-ST-elevation myocardial infarction; STEMI, ST-elevation myocardial infarction.

A two-sided *p*-value less than 0.05 is represented by bold numbers.

In the multivariate linear regression analysis, gender or age did not affect the number of hospital admissions due to hypertension, CCS or AMI. However, there was a higher admission rate of female patients with unstable angina pectoris after the pandemic. In summary, the results of the linear regression analysis are in line with the results of our descriptive analysis: a gender or age dependent difference in hospital admission rates cannot be observed in all conducted regression models (Table 2).

**Table 2:** Results from the multivariate linear regression analysis adjusted for age and sex.

	Hypertension		CCS	
	β (95% CI)	*p*-value	β (95% CI)	*p*-value
Age	−0.001 (−0.003–0.001)	0.59	−0.001 (−0.003–0.002)	0.59
Gender	0.03 (−0.04–0.1)	0.38	0.04 (−0.011–0.09)	0.38
Age	−0.001 (−0.002–0.001)	0.61	0.000 (−0.003–0.002)	0.71
Gender	0.03 (−0.034–0.01)	0.33	0.05 (−0.006–0.1)	0.08
Diabetes mellitus	0.07 (−0.008–1.53)	0.77	−0.08 (−0.13 to −0.33)	0.01
CKD	–	–	–	–
Age	−0.001 (−0.003–0.001)	0.59	−0.001 (−0.003–0.001)	0.42
Gender	0.032 (−0.034–0.099)	0.33	0.04 (−0.01–0.09)	0.12
Diabetes mellitus	–	–	–	–
CKD	0.01 (0.00–1.94)	**0** **.** **05**	0.22 (0.15–0.3)	**<0** **.** **001**
Age	−0.001 (−0.003–0.001)	0.59	−0.001 (−0.003–0.002)	0.59
Gender	0.03 (−0.03–0.1)	0.33	0.055 (−0.005–0.11)	0.32
Diabetes mellitus	0.27 (0.098–1.53)	0.67	−0.23 (−0.29 to −0.18)	**<0** **.** **001**
CKD	0.07 (−0.08–0.2)	0.35	0.39 (0.31–0.47)	**<0** **.** **001**
	UAP		NSTEMI/STEMI (AMI)	
	β (95% CI)	*p*-value	β (95% CI)	*p*-value
Age	0.001 (−0.002–0.004)	0.44	−0.001 (−0.003–0.002)	0.57
Gender	0.11 (0.04–0.19)	**0** **.** **003**	−0.023 (−0.095–0.49)	0.52
Age	0.001 (−0.002–0.004)	0.59	−0.001 (−0.003–0.002)	0.53
Gender	0.11 (0.045–0.19)	**0** **.** **002**	−0.021 (−0.094–0.05)	0.56
Diabetes mellitus	0.18 (0.099–2.7)	**<0** **.** **001**	−0.007 (−0.079–0.066)	0.86
CKD	–	–	–	–
Age	0.001 (−0.002–0.004)	0.66	0.000 (−0.002–0.003)	0.47
Gender	0.11 (0.04–0.18)	**0** **.** **003**	−0.025 (−0.096–0.046)	0.84
Diabetes mellitus	–	–	–	–
CKD	0.26 (0.16–0.36)	**<0** **.** **001**	0.18 (0.082–0.27)	**<0** **.** **001**
Age	0.001 (−0.002–0.004)	0.67	0.000 (−0.002–0.003)	0.89
Gender	0.11 (0.04–0.19)	**0** **.** **002**	−0.23 (−0.095–0.05)	0.53
Diabetes mellitus	0.06 (−0.06–0.18)	0.33	−0.24 (−0.097–0.48)	0.51
CKD	0.2 (0.06–0.36)	**0** **.** **006**	1.8 (0.086–0.27)	**<0** **.** **001**

In addition, diabetes mellitus and the presence of a chronic kidney disease was added as potentially confounding variables in the full model.

CCS, chronic coronary syndrome; UAP, unstable angina pectoris; NSTEMI, non-ST-elevation myocardial infarction; STEMI, ST-elevation myocardial infarction.

A two-sided *p*-value less than 0.05 is represented by bold numbers.

**Figure 1:**
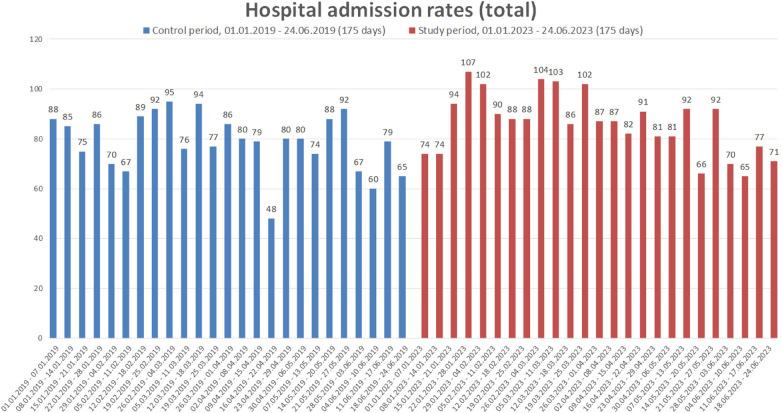
Weekly comparison of the total number of hospital admissions due to arterial hypertension, unstable angina pectoris, chronic coronary syndrome or acute myocardial infarction. Data are presented for whole weeks during the control period (01.01–24.06.2019) and study period (01.01–24.06.2023).

**Figure 2:**
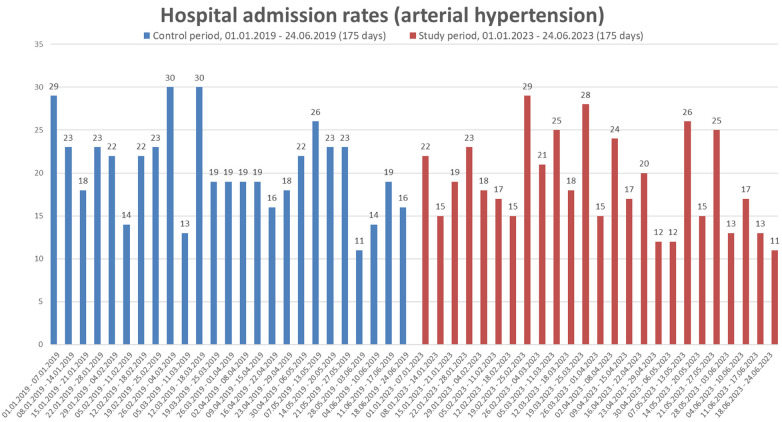
Weekly comparison of the total number of hospital admissions due to arterial hypertension. Data are presented for whole weeks during the control period (01.01–24.06.2019) and study period (01.01–24.06.2023).

A histogram comparing hospital admission rates on a weekly basis can be found as Figure 1 (total admissions rates) and Figure 2 (hospital admission rates for arterial hypertension). Possible differences between the control and study period regarding the distribution of weekly hospital admissions for arterial hypertension were further analyzed in a grouped week-by-week comparison using Mann–Whitney *U*-Test. Details can be found in Supplementary Table S4. In summary, no differences in hospitalization rates for high blood pressure were found between the control period and the study period (all *p* > 0.05).

Interestingly, the prevalence of comorbidities like chronic kidney disease, atrial fibrillation, and heart failure was slightly lower in patients with coronary artery disease in the post-pandemic era. As a contrast, with the exception of chronic kidney disease, there was no difference in co-morbidities compared to the control period in patients presenting with AMI. The distribution of comorbidities can be found in Table 3.

**Table 3:** Proportions of comorbidities for admitted patients during the control and study period.

	Hypertension			CCS		
	Control period	Study period	*p*-value	Control period	Study period	*p*-value
Total	516	483	0.07	759	943	**<0** **.** **001**
Diabetes mellitus	104 (20.1%)	76 (15.7%)	**0** **.** **004**	234 (30.8%)	259 (27.4%)	0.12
Lipids	156 (30.2%)	164 (33.9%)	0.2	490 (57.5%)	676 (71.9%)	**0** **.** **002**
Hypertension				640 (84.3%)	768 (81.4%)	0.11
Heart failure	52 (10.0%)	34 (7.0%)	0.08	265 (34.9%)	228 (24.1%)	**<0** **.** **001**
CKD	69 (13.3%)	45 (9.3%)	**0** **.** **04**	145 (19.1%)	83 (8.8%)	**<0** **.** **001**
AF	70 (13.5%)	57 (11.8%)	0.4	161 (21.2%)	155 (16.4%)	**0** **.** **002**
	UAP			NSTEMI/STEMI (AMI)		
	Control period	Study period	*p*-value	Control period	Study period	*p*-value
Total	270	451	**<0** **.** **001**	487	349	**<0** **.** **001**
Diabetes mellitus	85 (31.4%)	78 (17.2%)	**<0** **.** **001**	151 (31.0%)	111 (31.8%)	0.8
Lipids	134 (49.6%)	267 (59.2%)	**0** **.** **012**	280 (57.5%)	251 (71.9%)	**<0** **.** **001**
Hypertension	206 (76.2%)	347 (76.9%)	0.8	354 (72.6%)	266 (76.2%)	0.25
Heart failure	55 (20.3%)	101 (22.4%)	0.54	269 (55.2%)	175 (50.9%)	0.14
CKD	59 (21.8%)	37 (8.2%)	**<0** **.** **001**	161 (33.0%)	55 (15.7%)	**<0** **.** **001**
AF	46 (17.0%)	70 (15.5%)	0.59	129 (26.4%)	76 (21.7%)	0.11

Performed cardiac procedures and corresponding proportions of comorbidities during the control and study period.

CCS, chronic coronary syndrome; UAP, unstable angina pectoris; NSTEMI, non-ST-elevation myocardial infarction; STEMI, ST-elevation myocardial infarction; CKD, chronic kidney disease; AF, atrial fibrillation; PCI, percutaneous coronary intervention.

A two-sided *p*-value less than 0.05 is represented by bold numbers.

Rates of coronary angiographies conducted increased during the study period, while the proportion of PCI therapy insignificantly decreased at the same time (46.5% vs. 44.5%, *p* = 0.04). Age, gender distribution, duration of hospitalization and the proportions of comorbidities for patients undergoing cardiac procedures are summarized in Table 4.

**Table 4:** Performed cardiac procedures, age, gender and corresponding proportions of comorbidities during the control and study period.

	Coronary angiography			PCI		
	Control period	Study period	*p*-value	Control period	Study period	*p*-value
Total	2.248	2.579	**0** **.** **002**	1.046	1.150	0.49
Difference		+331 (14.7%)			+104 (9.9%)	
% performed PCIs	46.5%	44.5%	0.4			
Female	869 (38.7%)	1,050 (40.7%)	0.14	307 (29.3%)	369 (32.1%)	0.16
Age (years)	68.5 (±12.6)	69.5 (±11.7)	**0** **.** **013**	69.8 (±11.8)	71.2 (±10.9)	**0** **.** **003**
Length of stay (days)	4.2 (±4.9)	3.8 (±4.0)	**<0** **.** **001**	4.5 (±5.8)	4.0 (±4.1)	**<0** **.** **001**
Diabetes mellitus	622 (27.6%)	641 (24.8%)	**0** **.** **02**	331 (31.6%)	350 (30.4%)	0.54
Lipids	1.131 (50.3%)	1.487 (57.6%)	**<0** **.** **001**	652 (62.3%)	745 (64.7%)	0.23
Hypertension	1.445 (64.-3%)	1.710 (66.3%)	0.14	693 (66.2%)	789 (68.6%)	0.23
Heart failure	1.054 (46.9%)	1.063 (41.2%)	**<0** **.** **001**	465 (44.5%)	453 (39.3%)	**0** **.** **01**
CKD	496 (22.0%)	301 (11.6%)	**<0** **.** **001**	250 (23.9%)	151 (13.1%)	**<0** **.** **001**
AF	732 (32.5%)	721 (27.9%)	**<0** **.** **001**	282 (26.9%)	270 (23.4%)	0.06

CCS, chronic coronary syndrome; UAP, unstable angina pectoris; NSTEMI, non-ST-elevation myocardial infarction; STEMI, ST-elevation myocardial infarction; CKD, chronic kidney disease; AF, atrial fibrillation; PCI, percutaneous coronary intervention.

A two-sided *p*-value less than 0.05 is represented by bold numbers.

## Discussion

The present study for the first time investigated hospital admission rates for arterial hypertension and coronary heart disease as a surrogate parameter for cardiovascular risk before and after the SARS-CoV-2 pandemic. Compared to the pre-pandemic era, there was no increase in hospitalizations for arterial hypertension or myocardial infarction. Thus, there is no evidence that a potentially persisting endothelial damage after COVID-19 might have led to an increase in arterial hypertension or coronary events in this cohort. Interestingly, however, there was indeed an increased number of patients who presented with chest pain without any indication for acute or chronic myocardial ischemia. It may be speculated that patients with post-acute sequelae of COVID-19 (Long-Covid) might have contributed to this finding. Past studies reported persisting symptoms including chest pain and dyspnea within the general population previously infected with SARS-CoV-2 (4, 5). NO-dependent vasodilation as a marker of endothelial function can be measured by flow-mediated dilation. An impairment of flow-mediated dilation has been reported in small cohorts of convalescent COVID-19 patients, especially when residual clinical manifestations persist (6). It is therefore feasible that changes in vascular function are accountable for persisting long-term symptoms. Chest pain without any coronary macrovascular correlate is a rather frequent finding in Long-COVID and represents a characteristic example of ongoing symptoms without objective measures of impaired cardiopulmonary health (7). Moreover, Long-COVID can be associated the musculoskeletal complaints which may be misinterpreted as cardiac chest pain. Finally, many people avoided hospitalizations during the pandemic and might now be more prone to catch up diagnostic measures that they had missed before. In line with these hypotheses, the total number of coronary angiographies increased, while the total numbers of PCIs decreased, even if statistically not significant. Hence, the intervention rate was significantly higher before than after the pandemic.

Lockdowns and the fear of infections during the pandemic had an impact on the number of health care contacts, both in an in- and outpatient setting. In order to avoid variations due to downtime effects, we compared hospitalization rates before and after the pandemic—not during the pandemic. It cannot be excluded that longterm downtime effects may have led to compensatory increased healthcare contacts in the early post-pandemic period. If so, however, the present hospitalization rates would even overestimate the factual rates. Hence, our analysis provides indeed no indication for an increased post-pandemic cardiovascular risk.

Interestingly, patients diagnosed with CCS or UAP showed lower proportions of comorbidities (diabetes mellitus, heart failure, chronic kidney disease and atrial fibrillation). A similar observation can be found in cases of performed coronary angiography: the proportion of comorbidities was significantly lower, the patients in general healthier.

Regarding gender distribution, our results are in line with previous findings, suggesting higher rates for hospital admissions due to arterial hypertension or hypertensive crises in women and acute myocardial infarction for men (8, 9). Noteworthy, we found an increase in admission rates of female patients due to chest pain (UAP) after the pandemic (30.7%–42.4%, *p* = 0.002). Several studies reported female sex as an independent risk factor of persisting symptoms after a SARS-CoV-2 infection (10–12), indicating sex-differences in Long-COVID syndrome.

Interestingly, the duration of hospitalization significantly decreased over time in all groups. This finding might result from the efforts made to minimize the duration of hospitalization during the pandemic for reasons of capacity, e.g., by improved logistic processes. Data regarding the pandemics’ long term impact on the general duration of hospitalizations would be of high interest but are lacking so far.

Past studies conducted during the pandemic indicate that individuals who recovered from the first wave of the SARS-CoV-2 infection utilized health care resources more frequently (13), but it remains unclear, if this effect perseveres after the start of the vaccination campaign in early 2021. However, since the vast majority of people has been infected by SARS-CoV-2 in the meantime, it is almost impossible to perform any larger studies on this issue. Therefore, the present analysis is of especial clinical relevance: Even if there was a persisting endothelial damage, this damage does not increase cardiovascular morbidity as measured in hospitalizations for arterial hypertension or coronary artery disease.

The study is limited by its regional character and the way of data extraction based on documented diagnoses. We had no insight into the individual diagnostic pathways. Furthermore, most patients included in the study period are assumed to be less susceptible to SARS-CoV2 infection or severe COVID-19, and these patients who are likely to have mild COVID-19 would be similar to the general population. Thus the comparison between patients from the study period and the control period is not a balanced case-control design. This study is unable to identify deceased COVID-19 patients that may have more hypertension related diseases. It remains unclear, whether the high mortality rates during the early COVID-19 pandemic is reflected in the non-significant difference regarding hospital admission rates due to hypertension. On the other hand, it is a multicenter study encompassing three large hospitals in a German city region with a representative number of hospitalizations over 6 corresponding months prior and after the pandemic. Future studies will have the opportunity to examine longer periods after the end of the pandemic.

It has been speculated that SARS-CoV-2 infections might lead to persistently increased cardiovascular morbidity and mortality. The present multicenter study does not show an increase in hospitalizations for arterial hypertension or coronary artery disease before and after the pandemic as a surrogate parameter of cardiovascular risk.

## Data Availability

The data analyzed in this study is subject to the following licenses/restrictions: Hospital administrative data was analyzed for this manuscript. Anonymous datasets can be shared upon reasonable request. Requests to access these datasets should be directed to benjamin.sasko@elisabethgruppe.de.
