# Supplementary material for: Impact of the COVID-19 pandemic on hospital admission rates for arterial hypertension and coronary heart disease: a German database study
Source: Front Cardiovasc Med. 2024 May 14;11:1333749. doi: 10.3389/fcvm.2024.1333749 (PMC11135167; doi:10.3389/fcvm.2024.1333749)
Supplement: Supplementary file 1 [file Table1.docx]

| **Admission diagnose** | **ICD-10-code** |
| --- | --- |
| Arterial hypertension | I10.0, I10.1, I10.9 |
| Unstable Angina | I20.0, I20.1, I20.9 |
| Chronic coronary syndrome | I25.10, I25.11, I25.12, I25.13, I25.14, I25.15, I25.16, I25.19, I25.8 |
| Acute myocardial infarction | I21.0, I21.1, I21.2, I21.3, I21.4 |

**Supplementary Table 1:** ICD-10-codes of admission diagnoses

**Supplementary Table 2:** ICD-10-codes of comorbidities

| **Comorbidities** | **ICD-10-code** |
| --- | --- |
| Arterial hypertension | I10.0, I10.1, I10.9 |
| Diabetes mellitus | E10.01, E10.11, E10.20, E10.21, E10.30, E10.31, E10.40, E10.41, E10.50, E10.51, E10.60, E10.61, E10.72, E10.73, E10.74, E10.75, E10.80, E10.81, E10.90, E10.91  E11.01, E11.11, E11.20, E11.21, E11.30, E11.31, E11.40, E11.41, E11.50, E11.51, E11.60, E11.61, E11.72, E11.73, E11.74, E11.75, E11.80, E11.81, E11.90, E11.91 |
| Chronic kidney disease | N18.1, N18.2, N18.3, N18.4, N18.5, N18.89, N18.9 |
| Hypercholesterinemia | E78.0, E78.2, E78.3, E78.4, E78.5, E78.8 |
| Congestive heart failure | I50.0, I50.1 |
| Atrial fibrillation | I48.0, I48.1, I48.2, I48.9 |

**Supplementary Table 3:** OPS codes of cardiac procedures

| **Cardiac procedure** | **OPS code** |
| --- | --- |
| Coronary angiography | 1-278 |
| Percutaneous intervention | 8-837 |

**Supplementary Table 4:** Distribution of weekly hospital admissions for arterial hypertension during the control period and the study period. Data were analyzed by comparing admission rates of week 1 to 13 of the control period (285 admissions) with week 1 to 13 of the study period (265 submissions) using Mann-Whitney U-Test (p = 0.39). A similar analysis was performed by comparing admission rates of week 14 to 25 of the control period (226 admissions) with week 14 to 25 of the study period (205 submissions), also by using Mann-Whitney U-Test (p = 0.37). In summary, no differences in hospitalization rates for arterial hypertension were found in a grouped week-by-week comparison.

| **Week** | | **Admission rates** | | |
| --- | --- | --- | --- | --- |
|  | | **Control period (2019)** | **Study period (2023)** | **P value** |
| **1** | 01.01. - 07.01. | 29 | 22 | 0.39 |
| **2** | 08.01. - 14.01. | 23 | 15 |  |
| **3** | 15.01. - 21.01. | 18 | 19 |  |
| **4** | 22.01. - 28.01. | 23 | 23 |  |
| **5** | 29.01. - 04.02. | 22 | 18 |  |
| **6** | 05.02. - 11.02. | 14 | 17 |  |
| **7** | 12.02. - 18.02. | 22 | 15 |  |
| **8** | 19.02. - 25.02. | 23 | 29 |  |
| **9** | 26.02. - 04.03. | 30 | 21 |  |
| **10** | 05.03. - 11.03. | 13 | 25 |  |
| **11** | 12.03. - 18.03. | 30 | 18 |  |
| **12** | 19.03. - 25.03. | 19 | 28 |  |
| **13** | 26.03. - 01.04. | 19 | 15 |  |
|  | Total | 265 | 285 |  |
| **14** | 02.04. - 08.04. | 19 | 24 | 0.37 |
| **15** | 09.04. - 15.04. | 19 | 17 |  |
| **16** | 16.04. - 22.04. | 16 | 20 |  |
| **17** | 23.04. - 29.04. | 18 | 12 |  |
| **18** | 30.04. - 06.05. | 22 | 12 |  |
| **19** | 07.05. - 13.05. | 26 | 26 |  |
| **20** | 14.05. - 20.05. | 23 | 15 |  |
| **21** | 21.05. - 27.05. | 23 | 25 |  |
| **22** | 28.05. - 03.06. | 11 | 13 |  |
| **23** | 04.06. - 10.06. | 14 | 17 |  |
| **24** | 11.06. - 17.06. | 19 | 13 |  |
| **25** | 18.06. - 24.06. | 16 | 11 |  |
| **26** | 25.06. - 30.06. | Excluded* | Excluded* |  |
|  | Total | 226 | 205 |  |

*Week 26 was excluded to achieve comparability of whole weeks without outliners.
